# A Comparison of Otolaryngology Training in Five English-Speaking Countries

**DOI:** 10.7759/cureus.99249

**Published:** 2025-12-15

**Authors:** Fady Kamel, Amir Habeeb, John Fahmy, Pierre Elnazir, Haroon Khokher, Mohammed Sayed, Swastik Sutar, Hesham Kaddour

**Affiliations:** 1 General Surgery, West Suffolk Hospital, Bury St Edmunds, GBR; 2 Surgery, Health Education East of England, Norwich, GBR; 3 ENT Surgery, Barking, Havering and Redbridge University Hospitals NHS Trust, Romford, GBR; 4 General Medicine, Institute of Sports and Exercise Health, University College London, London, GBR; 5 Anatomy, King's College London, London, GBR; 6 ENT, Luton and Dunstable Hospital, Luton, GBR; 7 Trauma and Orthopaedics, Peterborough City Hospital, Peterborough, GBR

**Keywords:** comparison, countries, ent, international medical graduates, otolaryngology, training

## Abstract

Otolaryngology was conceived at the turn of the twentieth century as a product of the amalgamation of the separate disciplines of the primarily surgeon-led otology and physician-led laryngology. Since its conception, otolaryngology has flourished and continues to, due to the many advancements in medical technologies. We aim in this review to provide a comparison between the postgraduate training pathway for otolaryngology in five English-speaking countries, highlighting the main differences, strengths, and drawbacks of each pathway. This, we hope, will be able to guide future changes in the training pathway and inform trainees considering a career in otolaryngology overseas. Data on training programme, its pathway, duration, examinations, competition levels, and overseas applications were collected from literature, official governing bodies’ publicly available documents and online resources.

Otolaryngology training pathways differ between the United Kingdom (UK), the United States of America (USA), Canada, and Australasia. The differences are highlighted in the structure, duration, and assessment and have been adapted to reflect each nation’s healthcare system and educational priorities. The UK uses an outcome-based model with emphasis on broad surgical exposure before specialisation, whereas the USA offers a shorter direct entry into a five-year residency programme with a focus on high procedural volume. Canada blends both the USA’s residency structure with the UK’s outcome-based approach to ensure consistency of knowledge and skills across its diverse training network. Australasia adopt a three-staged competency framework which allows its trainees to qualify and practice in both nations. International medical graduates (IMGs) across all five countries face many barriers, ranging from tiered systems entry in the UK and Australasia, to highly restrictive processes in Canada and low match rates in the USA.

Despite variations, all systems aim to produce competent, independent consultants through rigorous application pathways, competency-based training, national examinations, and subspeciality exposure. Surgical training is adapting to increasing emphasis on cultural competence and professional behaviours.

## Introduction and background

Introduction

Otolaryngology emerged at the beginning of the twentieth century as a fusion of two distinct fields: the largely surgeon-driven otology and the physician-led laryngology [1]. Since its inception, otolaryngology has thrived, propelled by numerous advancements in medical technology, such as the introduction of the Hopkins endoscope in the 1960s and the adoption of operating microscopes [1,2]. More recent innovations include robotic surgery, which enables complex surgical procedures using minimally invasive techniques [3]. Furthermore, otolaryngology encompasses a wide range of subspecialities, catering to the diverse interests of its trainees. These include otology, otoneurosurgery and skull-base surgery, head and neck surgery, phonosurgery, rhinology and facial plastics, and paediatric otolaryngology [4].

This review aims to study and compare the postgraduate training pathways for otolaryngology across five English-speaking countries with well-established, reputable programs: the United Kingdom (UK), the United States of America (USA), Canada, Australia, and New Zealand. We analyse the key differences, strengths, and challenges of each system, with the goal of guiding future reforms in training pathways and offering valuable insights for trainees considering a career in otolaryngology.

Methods and materials

Data was gathered from literature, publicly accessible documents from official governing bodies, and online resources. The primary variables analysed included the structure of the training pathway, competition rates, curriculum content, program duration, examination requirements, and the process for overseas candidates to enter the training program.

## Review

United Kingdom (UK)

Training Pathway

All newly qualified graduates in the UK need to complete the common two-year foundation training pathway: Foundation Year 1 (FY1) and Foundation Year 2 (FY2). A full General Medical Council (GMC) license is obtained upon the completion of the FY1 [5]. Doctors wishing to apply for surgical training traditionally are required to competitively apply for a two-year Core Surgical Training (CST) post and undertake the Multi-Speciality Recruitment Assessment (MSRA) [6]. This constitutes phase 1 of the three phases in otolaryngology training in the UK. Phases 2 and 3 involve the development of all the knowledge and skills needed to qualify as a consultant in general otolaryngology and developing further skills, including the technical competencies in a special subarea of interest, respectively [7].

During the two years of CST, trainees rotate every 4-6 months between an array of surgical specialities and related ones. All prospective ENT applicants will need to obtain a minimum of six months of otolaryngology experience, although it is recommended to aim for 12 months. Candidates are required to sit examinations in order to obtain Membership of the Royal College of Surgeons (MRCS) and a Diploma in Otolaryngology-Head and Neck Surgery (DO-HNS) within two years of CST. From February 2026, applicants will be required to obtain the general intercollegiate MRCS and not the MRCS (ENT). This would take place before applying for speciality training in the case of the CST programme [4,6-8]. In 2018, some deaneries had started offering a run-through pathway for candidates who had decided early on to pursue a career in otolaryngology. This means they would have had to competitively apply for specialised otolaryngology training and, if successful, be appointed as a speciality trainee 1 (ST1), where they will follow an otolaryngology-themed programme for two years and carry on to higher speciality training without the need to competitively apply after the first two years, as is the case for CST. This pathway no longer exists [6-8]. Some candidates with an interest in academic medicine can apply to the Academic Clinical Fellowships (ACFs) in Otolaryngology. These pathways are available as “run-through” after successful completion of FY2 or through national selection at ST3 level after successful completion of CST. ACF trainees are allocated 75% clinical time and 25% academic time. Many of these candidates use this time to pursue academic commitments with the goal of generating pilot data for a PhD application [4].

Higher speciality training in otolaryngology is six years long (ST3-ST8). Candidates need to apply separately to this after completion of the two CST years. This makes the total duration of ENT training 10 years after medical school. By the end of those six years, trainees need to have sat and passed the intercollegiate speciality examination (FRCS) in order to obtain the Certificate of Completion of Training (CCT). Although not a formal part of training, many trainees choose to undertake a post-CCT or within-training fellowship in an area of their interest. These fellowships are usually overseas in specialised centres that offer a unique experience that a trainee might find useful for their development. This might also help improve their job prospects in a certain country or in a certain subspeciality [5,6,8].

Competition

Surgery and otolaryngology are both very competitive. There were 5.25 applicants per place for a CST job in 2024, while there were 2.93 applicants per place for an otolaryngology ST3 job in the same year. The ratio of applicants per place fell from 2013 to 2016 from approximately 3:1 to 1.5:1. This ratio has been slowly rising and has reached 2.57:1 in 2019 before jumping to 5.7:1 in 2020. This has then dropped to 4.43 in 2021, 3.6 in 2022, and 2.93 in 2024. Due to the highly competitive nature of surgical specialities, applicants need to improve their portfolio to increase their likelihood of success. The selection process and the panels favour applicants who have shown commitment and interest in the speciality. Here are some of the ways applicants can prove this commitment: a medical elective/taster week that is completed in that speciality is beneficial, research and quality improvement projects related to the speciality or surgery in general, attending surgical courses, research publications, involvement in teaching and having an up-to-date surgical logbook [9-12].

Moving to the UK

Any international medical graduate (IMG) wishing to work in the UK will need a full GMC license [13]. Doctors from the European Economic Area (EEA) could previously have been eligible for a full GMC license if they had completed their basic medical training. However, this privilege of automatic recognition no longer applies since the first of January 2021, when the UK left the EEA [14]. All IMGs now will need to satisfy the following criteria to obtain the full GMC license: demonstrate the GMC’s English language requirements by scoring a minimum of 7.5 in the academic version of the International English Language Testing System (IELTS) test (or pass the Occupational English Test at level B), and pass the two-part Professional and Linguistic Assessment Board (PLAB). The first part of the PLAB can be done overseas, but the second part needs to be done in the UK [15].

IMGs who have completed either FY1 in the UK or an equivalent period of training overseas will be able to apply for a full GMC license. Those who have not will only be able to apply for a provisional one and undertake the FY1 year in order to be able to apply for a full license [13].

Alternatively, for IMGs who have completed training abroad in a non-approved training programme by the GMC, they can be entered into the specialist register by obtaining a Certificate of Eligibility for Specialist Registration (CESR). Applicants will need to demonstrate in their portfolio that they have satisfied the training and experience requirements of the otolaryngology CCT curriculum. Once the candidate has obtained their CESR, they are exempt from the PLAB exam [16].

Training Requirements

To complete ENT training in the UK and obtain the CCT, trainees must follow a structured pathway through the Joint Committee on Surgical Training (JCST) curriculum. Higher speciality training can be split into three phases. During phase 1 (ST3-ST4), trainees gain basic knowledge and surgical skills across all ENT subspecialities, while in phase 2, trainees gain more advanced training in general ENT and subspeciality areas. In phase 3, trainees focus on consolidating skills, with opportunities to specialise in specific areas like otology, rhinology, head and neck, paediatrics, or thyroid surgery. Trainees must prove their proficiency through completing workplace-based assessments (WBAs) and Multiple Consultant Report (MCR), leading to the Annual Review of Competence Progression (ARCP). The new curriculum that was introduced in 2021 shifted the emphasis away from a pure number of procedures undertaken to an outcome-based training system. Trainees must also demonstrate progression with capabilities in practice (CIPs) such as outpatient management, theatre list management, and emergency care. Trainees must pass the Fellowship of the Royal College of Surgeons exam in otolaryngology, which is typically undertaken in ST7-ST8, to obtain the CCT [17-19].

**Figure 1:**
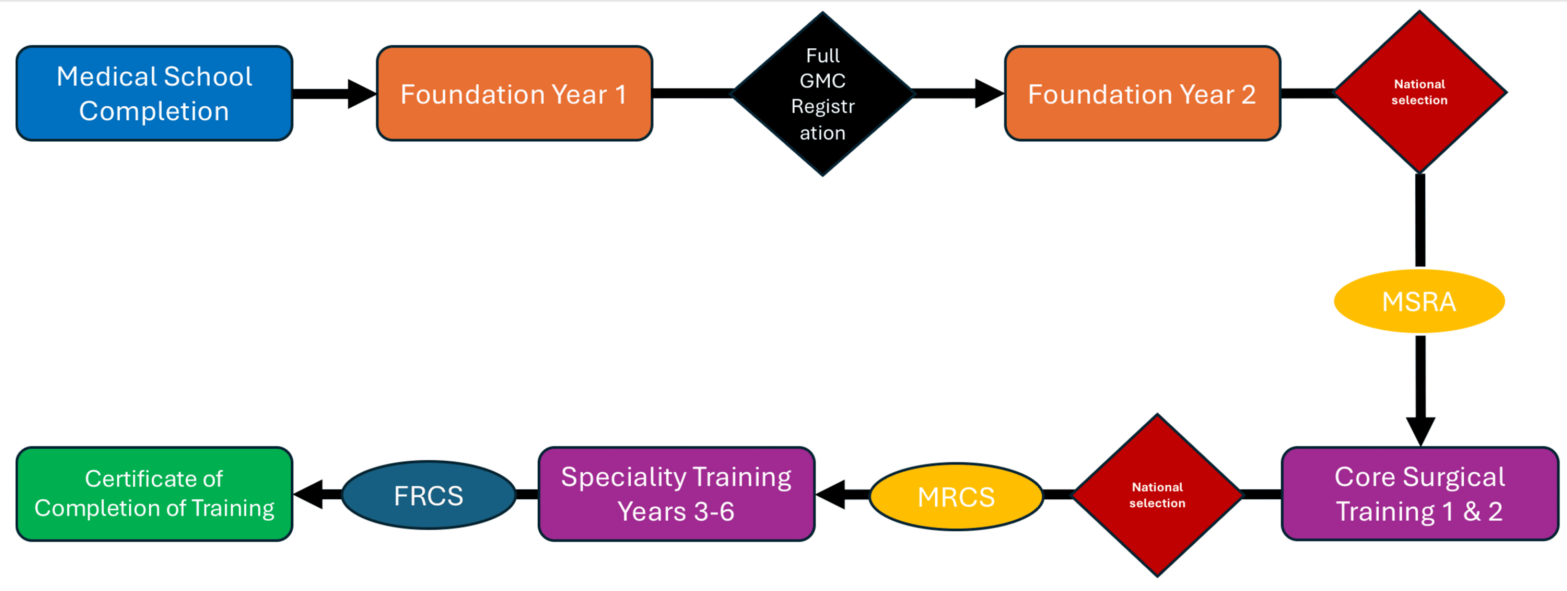
This diagram outlines a summary of the pathway for UK graduates to train in otolaryngology

The pathway is rigorous and designed to ensure that trainees are competent and confident to perform as independent consultants by the time they finish. Furthermore, regular feedback and meetings with educational supervisors help tailor the learning process to individual needs throughout the training. Consultants maintain their license to practice by conducting regular revalidations with the GMC [17-19]. Refer to Figure 1 for a summary of the outline and key features of the UK training program.

United States of America (USA)

Training Pathway

Following the completion of an undergraduate and a medical degree, graduates undertake a five-year residency training programme, which is accredited by the Accreditation Council for Graduate Medical Education (ACGME). Applicants for training in otolaryngology are allocated places through the National Resident Matching Process (NRMP). Following completion of the residency training programme, trainees have the option of choosing to pursue a fellowship training programme for two years in an ENT subspeciality of their choice. In terms of exams and certification, all medical graduates in the USA who wish to practice medicine must pass the United States Medical Licensing Exam (USMLE) steps 1, 2, and 3 in order to obtain a license to practice. Those who undertake a residency in otolaryngology have the option to apply for board certification from the American Board of Otolaryngology. This would need to be renewed every few years, depending on the state [20-22].

Competition

Otolaryngology training competition in the USA remains very high; this is due to the very strong pool of applicants. Many unsuccessful applicants reapply for an otolaryngology residency with a good success rate [23,24]. There were 361 places available in 2023 for 556 applicants; this is a rate of 1.54 applicants per place [19]. Due to this relatively high competition, applicants need a well-balanced and strong application to prove their commitment and competency. Applicants with high grades on their otolaryngology rotations in medical school are at an advantage. In addition, above-average scores in the USMLE (230-240) are highly advantageous. The successful undertaking of research projects related to otolaryngology, or surgery in general, demonstrates a genuine interest in the speciality and is highly desirable [25,26].

Moving to the USA

IMGs wishing to practice medicine in the USA will require a certification from the Educational Commission for Foreign Medical Graduates (ECFMG). In order to obtain this, IMGs will need proof of successful completion of a medical school and to sit and pass steps 1 and 2 of the USMLE. Once they have been certified, they can then apply for residency through the NRMP. Unfortunately, the match rate for IMGs for a residency in otolaryngology is very low. In 2025, seven of the places filled were allocated to IMG applicants [19,25,27]. Furthermore, there are additional hurdles that IMGs face compared to their US counterparts; this includes obtaining local letters of recommendation and a good local clinical experience [25,27].

Training Requirements

To complete their training, residents must obtain the American Board of Otolaryngology-Head and Neck Surgery (ABOHNS) certification. Residents must follow a rigorous, structured pathway which includes one year of general surgery training (PGY1) and four years of otolaryngology-specific training (PGY2 to PGY5). Residents must demonstrate their clinical competency during their annual evaluation based on the otolaryngology milestones. These track the development of their knowledge, skills, surgical techniques, and professional portfolio development. Developing competence in areas like head and neck surgery, otology, rhinology, and facial plastic surgery is critical. Residents must also seek to complete a minimum number of logged surgical cases, which is set out by the ACGME across a variety of categories, including airway surgeries, head and neck surgeries, paediatric ENT procedures, and otology-based surgeries. Residents are required to undertake otolaryngology in-training examinations (OITE) annually to test their theoretical knowledge. This can aid in the outlining of areas where the resident may need to improve. After completing the residency program, residents must pass the ABOHNS exams, both written and oral, certifying exams to gain board certification. While not a requirement, some residents may wish to undertake a fellowship to further subspecialise in different areas [19,28,29].

Canada

Training Pathway

Applicants for training in otolaryngology are allocated places through the Canadian Residency Matching Service (CaRMS). Candidates who wish to specialise in otolaryngology need to complete a five-year residency programme. They can join directly after graduation from medical school. The first two years are a foundational training in surgery, split into 26 blocks (each block is roughly equivalent to four weeks). Between 10 and 18 of those blocks are designated for OHNS and related specialities. For a minimum of three and a maximum of six blocks, trainees rotate between different surgical specialities, for example, Plastic Surgery, Neurosurgery and General Surgery. This allows them to acquire the baseline surgical knowledge and skills needed to practice surgery. Trainees can then choose from a variety of other allied specialities: paediatrics and paediatric surgery, thoracic surgery, internal medicine, oncology and oral and maxillofacial surgery, for a further six-block rotation, doing 1-2 blocks per speciality. In addition, they are also required to briefly undertake a rotation in trauma-related speciality (emergency medicine, plastic surgery or general surgery), critical care medicine, and anaesthesiology. Trainees can also undertake a maximum of six blocks in otolaryngology-related research [30].

On completion of residency, trainees must pass the Royal College certification examination in OHNS, which consists of a written and applied component. Successful candidates are then eligible to practice independently and obtain provisional licensure. Many graduates choose to pursue fellowship training to consolidate subspeciality expertise [31,32].

Competition

Otolaryngology is consistently one of the most competitive surgical specialities in Canada. The rate of applicants to the number of places filled is higher than the average across all surgical specialities. The application figures are not routinely published; however, in 2025, there were 36 available spaces. CaRMS reports routinely categorise otolaryngology within the group where demand exceeds supply for Canadian medical graduates [33].

Moving to Canada

There are differences in the process of licensure between provinces and territories in Canada. Essentially, all IMGs need to pass the Medical Council of Canada Evaluating Examination (MCCEE) parts 1 and 2 and the National Assessment Collaboration Examination (NAC). If required, they need to demonstrate their proficiency in the English language by achieving a minimum score of 7 in the IELTS exam. Additionally, IMGs need to be permanent residents or citizens of Canada. Having achieved the previous requirements, IMGs can then apply for residency through CaRMS. Meanwhile, IMGs who have completed speciality training outside of Canada and the USA can obtain a license through another route, the “approved jurisdiction” route. The Royal College of Physicians and Surgeons of Canada (RCPSC) certifies its training if they were trained in one of the international jurisdictions that are deemed to have met the standards of the RCPSC. The UK, Australia, New Zealand, South Africa, Switzerland, Singapore, Hong Kong, and Ireland are all approved jurisdictions. IMGs will then need to pass the Royal College exams in order to be certified in their speciality. As reflected in all these criteria, the process of IMGs moving to work in Canada and obtaining a license to practice medicine is by far the most difficult one out of the five countries in this review [34-37].

Training Requirements

Canadian residency has transitioned to Competence by Design (CBD) since 2019. This is a competency-based model developed by the RCPSC, which emphasises staged outcomes-based progression. This occurs through four phases of training: transition to discipline, foundations, core, and transition to practice. All these competencies are mapped to CanMEDS roles (an educational framework that outlines competencies needed by physicians to effectively meet the needs of the population) and defined through entrustable professional activities (EPAs). These EPAs, such as assessing acute airway obstruction, managing epistaxis, performing nasal endoscopy, and treating deep neck space infection, are observable tasks that residents must demonstrate repeatedly to a standard allowing entrustment for independent performance. The progress of such tasks is monitored through WBA, structured feedback and review by the programme competency committee, with a final sign-off from the programme director. OHNS was one of the first surgical specialities to adopt this model of competency-based structure in Canada. Its design is hailed to ensure graduates are prepared for independent consultant practice [38-44].

Early training also incorporates preparation for the Surgical Foundation examination after the first year of training, before embarking on senior OHNS training. To be eligible for the Royal College certification examination, the trainee must be enrolled in an accredited programme, complete all EPAs and curricular requirements, receive eligibility to examine ruling from the Royal College, and pass both the written and applied components of the final exam [38-44].

Australia and New Zealand

Training Pathway

Otolaryngology training in both Australia and New Zealand is delivered through the Surgical Education and Training (SET) by the RACS in collaboration with the Australian Society of Otolaryngology Head and Neck Surgery (ASOHNS) and the New Zealand Society of Otolaryngology Head and Neck Surgery (NZSOHNS) [45-46].

Applicants who wish to apply for OHNS are usually expected to apply during their third postgraduate year, after completing PGY1 and 2, to ensure that they achieve all the required competencies and prerequisites in both Australia and New Zealand. The SET requires them to meet a large number of eligibility criteria, including sitting and passing the Royal Australasian College of Surgeons (RACS) Generic Surgical Sciences Exam (GSSE) and sitting the new pilot Situational Judgement Test (SJT) from 2021 onwards (the SJT will not be used to determine the selection outcome). Applicants must also complete mandatory preparatory modules such as 'operating with respect' and 'cultural safety training', with New Zealand applicants undertaking additional Māori health and cultural competency modules [45-50].

Completion of training requires satisfactory sign-off at the competent stage and passing the Fellowship Examination in OHNS, which includes written and clinical components. Successful graduates are awarded Fellowship of the Royal Australasian College of Surgeons (FRACS) in OHNS, enabling specialist consultant practice in both Australia and New Zealand, with mutual recognition across jurisdictions [45,48,51].

Competition

The competition rates for OHNS are very high in Australia. The published figures for the trainees selected into the SET program for otolaryngology in 2024 were 65 applicants to 22 places; this is a ratio of 2.95 applicants per place [52].

The competition rates for OHNS are very high in New Zealand. The published figures for the trainees selected into the SET program for otolaryngology in 2024 were 17 applicants to four places; this is a ratio of 4.25 applicants per place [52,53].

Moving to Australia and New Zealand

There are three routes available for IMGs (who obtained their medical degrees outside of Australia and New Zealand) to practice in Australia: the standard pathway, the competent authority pathway, or the specialist pathway. All pathways require IMGs to meet the English language proficiency standards (IELTS Academic overall ≥7.0 or OET B in all components) [54].

IMGs from the UK, USA, Canada, and Ireland who have completed their primary medical qualification can apply for a provisional registration with the Medical Board of Australia through the competent authority pathway. Those IMGs can obtain a full general registration after the successful completion of 12 months (minimum 47 weeks) of supervised practice to get acquainted with medical practice in Australia. After obtaining the full general registration, IMGs can then apply for otolaryngology speciality training [54].

IMGs who are not eligible for the competent authority pathway can apply through the standard pathway. A primary medical qualification from an institute that is recognised by the Australian Medical Council (AMC) and the World Directory of Medical Schools is required in order to start the application process. Following the initial application for assessment, most IMGs will need to undertake the AMC Computer Adaptive Test (CAT) MCQ before completing 12 months (minimum 47 weeks) of supervised practice to get acquainted with medical practice in Australia [54].

IMGs who have completed their otolaryngology speciality training and have been certified in their own countries can apply through the specialist pathway. An initial application is sent to the AMC for initial verification of the qualifications. This pathway is intended for fully qualified otolaryngology specialists. Applicants are evaluated for compatibility with the Fellow of the Australian Royal Australasian College of Surgeons standards. Those deemed substantially comparable can obtain provisional specialist registration and complete any required upskilling before full recognition; partially comparable applicants must undertake further training and assessment, while those found not comparable must enter training from the beginning [54].

In New Zealand, the IMG pathway is regulated by the Medical Council of New Zealand (MCNZ) and differs from its Australian counterparts in terms of structure and terminology. The most common route of entry is for training-level applications and is the comparable health system pathway. This system allows doctors trained in countries with similar medical and regulatory standards (UK, USA, Canada, Ireland, and Australia) to obtain provisional general registration after a short period of supervised practice in a New Zealand hospital. For those who trained in noncomparable systems, they are required to undertake the New Zealand Registration Examination (NZREX) through the NZREX clinical pathway before undertaking supervised practice. Alternatively, otolaryngology specialists can apply via the vocational pathway, with their comparability assessed by the RACS training board on behalf of MCNZ. Unlike Australia, there is no equivalent AMC written and clinical examinations for comparable health system applications, and the emphasis is largely on local supervision and culture competency training, particularly in Māori health [54].

Training Requirements

The programme follows a competency-based framework structured into three stages: novice, intermediate and competent. Progression occurs after demonstration of achievement of defined competencies and completion of EPAs across the breadth of subspecialities, including OHNS, laryngology, facial plastics, and paediatric ENT. These EPAs must be performed and assessed repeatedly to a standard that allows independent practice [55-57]

Assessment is continuous, incorporating workplace-based evaluation, surgical logbook reviews, end-of-term assessments (EOTAs), feedback, and consultant reports. The final progression decision is made by the RACS training board in consultation with the programme director [2-4]. To be awarded the FRACS, trainees in both countries must complete all required competencies and achieve satisfactory sign-off at the competent stage for all EPAs and pass the Fellowship Examinations, as stated previously. The structure of the training is designed to ensure that graduates are fully prepared for independent consultant practice in Australasia with mutual recognition across both jurisdictions [51,55-60]. 

Discussion

OHNS training in the UK, Australasia, Canada, and the United States differs in ways that reflect the organisation and incentives of each healthcare system and regulators. The UK’s lengthy, staged pathway evolved to serve a centrally planned National Health Service (NHS). The NHS prioritises equitable distribution of care and general productivity. Training is competency-based, following a strict 48-hour duty limit per week. This limits sheer operative volume, shifting assurances of readiness towards structured assessment and simulation while prioritising safety and well-being. Intercollegiate exist exam are key to the completion of training. Australasia operate a binational, college-led, five-year SET programme with a traditional final fellowship exam. The programme differs from that of the UK as it is less restricted by nationally uniform duty hour constraints, and greater local discretion, features that suit geographically relevant services, smaller trainee cohorts and a stronger apprenticeship model and culture. Canada’s programme is largely competence-by-design (EPAs/milestones) led. This standardises outcomes across diverse provincial systems. Duty hours tend to differ based on provinces, which allows for somewhat more flexibility compared to strict national caps. The United States blends a time-based five-year residency programme with an average of 80-hour limit weeks, milestones, and detailed case log minima. This structure tends to reflect a fragmented, insurance-driven environment needing strong external accreditation and a medicolegal culture that favours demonstrable metrics and exposure-driven supervision.

These large differences reflect the different educational philosophies across English-speaking countries. The Commonwealth system historically favours an apprenticeship-based system in addition to a rigorous exam culture (MRCS, FRCS, Royal College Accreditation). These systems rely on longitudinal faculty judgement and competency-based assessment to certify readiness. North American programmes tend to lean more towards numerical accountability and generally have a more pragmatic assessment culture. They rely on frequent milestone reviews and high-volume subspeciality exposure. The diversity across these designs highlights the fact that none of these designs is inherently superior. Each of these systems is an adaptation of its service model.

Selection for training remains one of the most competitive across surgical specialities across the five English-speaking countries in this review. Selection processes vary across countries based on the level of entry into training. The UK and Australasia emphasise the importance of portfolios and the use of structured interviews as a key factor in the selection process. The United States and Canada focus on programme fit, research and matching nationally using centralised exams. Less than full-time (LTFT) pathways and general well-being policies are much more developed in the UK compared to the other countries included in this review.

Career trajectories tend to mirror training philosophies and labour markets. The UK and Australasia produce generalists who gain subspeciality depth via one- to two-year fellowships. United States graduates often train in their chosen subspeciality early on, and Canada tends to occupy a middle ground. Research expectations range from protected, high-output models such as the United States model to service-integrated quality improvement and teaching in the UK and Australasia.

For IMGs, the barriers to entry vary widely between the countries examined in this review. The UK and Australasia have tiered pathways based on the applicant’s country of qualification. Canada’s IMGs have to endure the most restrictive entry requirements, which also vary between provinces and strongly prefer the Canadian medical graduates. While the United States offers a standardised entry through the USMLE and NRMP, otolaryngology remains one of the most competitive specialities for IMGs with consistently low match rates.

## Conclusions

While otolaryngology training in all five countries aims to produce highly skilled, independent consultants, the structure, duration and entry requirements vary considerably. Each training pathway reflects the respective country’s health system priorities. Despite the differences in approaches to training, common trends emerge. All systems are moving towards a competency-based model, use national-level examinations for certification, and emphasise subspeciality exposure. There is also an increasing recognition of the importance of cultural competence, professional behaviour, and patient safety among surgeons, and this has been incorporated in the training curricula.

IMGs wanting to pursue a career in otolaryngology will often find themselves fighting difficult odds given the competitive nature of the speciality. In the UK, Australia, and New Zealand, there is a relatively more structured and transparent entry route, especially from recognised jurisdictions. Canada and the USA remain more challenging markets to enter.

Future reforms should focus on further implementation of the competency framework internationally and work on increasing the transparency and centralisation of the selection process. Cross-country collaborations, exchange fellowships, and shared competency definitions can enhance global mobility among otolaryngology specialists and help align training with the evolving needs of modern healthcare systems.
